# Results following surgical resection of recurrent chordoma of the spine: experience in a single institution

**DOI:** 10.1186/s12957-020-02005-4

**Published:** 2020-08-27

**Authors:** Pongsthorn Chanplakorn, Thamrong Lertudomphonwanit, Wittawat Homcharoen, Prakrit Suwanpramote, Wichien Laohacharoensombat

**Affiliations:** 1 Department of Orthopedic, Faculty of Medicine Ramathibodi Hospital, Mahidol University, 270, Rama VI Road, Ratchathewi, Bangkok, 10400 Thailand; 2 Department of Orthopedics, Prapokklao Hospital, 38 Leab Noen Rd, Tambon Wat Mai, Mueang Chanthaburi District, Chanthaburi, 22000 Thailand

**Keywords:** Chordoma, Recurrence, Surgical resection, Radiotherapy, Oncologic outcome

## Abstract

**Background:**

Chordoma of the spine is a low-grade malignant tumor with vague and indolent symptoms; thus, large tumor mass is encountered at the time of diagnosis in almost cases and makes it difficult for en-bloc free-margin resection. Salvage therapy for recurrent chordoma is very challenging due to its relentless nature and refractory to adjuvant therapies. The aim of this present study was to report the oncologic outcome following surgical resection of chordoma of the spine.

**Materials and methods:**

Retrospective review of 10 consecutive cases of recurrent chordoma patients who underwent surgical treatment between 2003 and 2018 at one tertiary-care center was conducted**.**

**Results:**

There were 10 patients; 4 females and 6 males were included in this study. Eight patients had local recurrence. The recurrence was encountered at the muscle, surrounding soft tissue, and remaining bony structure. Distant metastases were found in 2 patients. The median time to recurrence or metastasis was 30 months after first surgery.

**Conclusion:**

En-bloc free-margin resection is mandatory to prevent recurrence. The clinical vigilance and investigation to identify tumor recurrent should be performed every 3 to 6 months, especially in the first 30 months and annually thereafter. Detection of recurrent in early stage with a small mass may be the best chance to perform an en-bloc margin-free resection to prevent further recurrence.

## Introduction

Chordomas are relatively rare, slow-growing, primary malignant bone tumors and comprise 17.5% of axial primary malignant bone tumors [1]. Because of their indolent and low-grade nature, chordoma is typically diagnosed at a late stage and therefore, often cause significant damage and compromise neurologic structures. The goal of treatment is to achieve surgical en-bloc excision with tumor-free margins to maximize local tumor control and overall survival but sometimes difficult to achieve this goal because of the complex surgical strategies and massive blood loss. Therefore, the rate or recurrent is high after the first surgery [2–5].

Local recurrence is the most important determinant of long-term survival, as Bergh et al. [4] reported a 21-fold increase in risk of tumor-related death in those with local recurrence, which greatly increased in an intralesional excision compared with en-bloc margin-free resection as confirmed in many studies [2–6]. However, wide en-bloc resection is not always possible, either because of the size or extent of the tumor or because such resection would lead to excessive morbidity. In this circumstance, the radiotherapy could play a major role for local control [7, 8].

The first treatment guideline for locally recurrent chordoma has been proposed by Chordoma Global Consensus Group in 2017 [9] and recommended the surgical treatment as one of the option, if possible, determined by the surgical plane, surrounding soft tissue, disability, comorbidity, and expected survival especially when high-dose radiation is not possible or available. Thus, the aim of this study was to report our experience in the treatment of recurrent chordoma. The surgical treatment strategies, location, and time of recurrence after each surgery and the oncologic outcome had also been assessed.

## Materials and methods

Institutional Review Board approval was obtained at our university medical center (COA.NO.MURA 2018/877). A retrospective review was conducted; the patients who were diagnosed for chordoma of the spine and underwent surgical treatment by two senior spine surgeons between 2003 and 2018 at our institution were enrolled in this study. The exclusion criteria were (1) patients with incomplete data or imaging and (2) had less than 1 year follow-up.

**Fig. 1:**
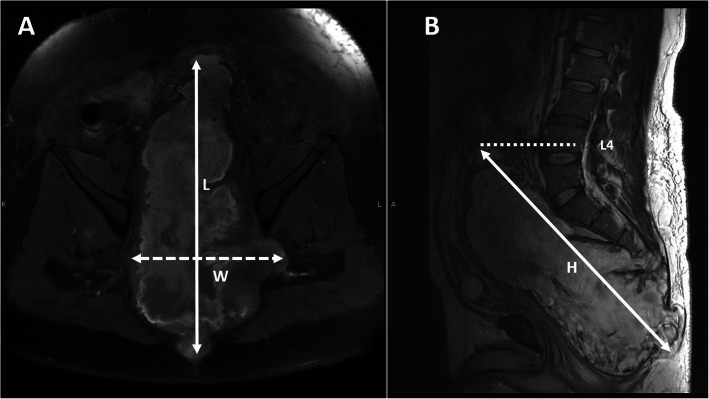
Measurement method, maximum length in axial image (**a**) on anterior-posterior (AP) dimension; *L,* in coronal dimension; *W*, and in sagittal reconstruction image (**b**); *H*, as illustrated in white arrow. The proximal vertebral level is defined as the upper most vertebrae at the end of the tumor mass (dot line in **b**)

Demographic data including age, sex, and location of tumor and center and proximal vertebral level of the tumor were collected. Mass size (maximum length in axial image on anterior-posterior (AP) dimension; *W*, coronal dimension; *L*, and in sagittal reconstruction image; *H*) was measured using Picture Archiving and Communication System (PACS) from magnetic resonance image (MRI) or computerized tomography scan (CT) images (Fig. 1). All patients had histological confirmation of chordoma of spine. Time of recurrence, recurrent pattern, type of the first index operation, and complications including death were collected from medical records. The recurrence of chordoma in this study was defined as the detection of tumor mass assessed by either MRI or CT scan.

## Results

### Patient characteristics

There were 10 patients; six males and four females, included in this present study. The primary location was at the sacrum in seven patients. One patient had the primary location at cervical spine. The other two patients had metastasis diseases, one patient was diagnosed at the initial visit and the other patient discovered at the time of follow-up period. The first index operation was performed at our hospital for all patients. Postoperative follow-up revealed local recurrences, by periodic CT or MRI. The diagnosis of chordoma in all patients was confirmed by a pathologist in our institution. The primary tumor sites, maximum tumor diameter in longitudinal transverse, and axial plane were measured by CT or MRI (Tables 1 and 2).

**Table 1:** Cases details of sacral chordoma

Case	Age	Sex	Mass size (*W* × *H* × *L*)	Primary location*	Operation	Reconstruction	Surgery (m/Yr)	Recurrent (month)	Complications
				Recurrent site					
Sacrum									
1	62	M	8.7 × 8.7 × 8.7	S4/S3	Excision S3		***August /2014***		
			5.3 × 6.0 × 4.2	Ischium	Excision		July/2018	11	
			6.1 × 4.3 × 3.7	Surgical bed	Excision		March/2019	7	Rectal tear
2	45	M	14.9 × 18.7 × 23.6	S4/L4	Debulking		***December/2015***		
			7 × 7 × 7	S3/S2	Excision S3		September/2018	33	seroma
3	57	F	11.4 × 9.0 × 7.6	S4/S2	Excision S2		***July/2009***		
			0.5–3^+^	Surgical bed	Excision mass		June/2011	20	
			5.8 × 2.5 × 3.8	Lt SI joint	Lt S1 hemiresection	Fibular strut	June/2013	21	
4	54	M	10.6 × 10 × 13	S3/S2	Excision S2		***February/2010***		
			11.3 × 8.3 × 4.2	S1 and Rt SI joint and Rt gluteal muscle	Tumor removal (S1+ SI joints resection)		January/2013	34	Massive bleeding
5	45	F	8.7 × 11.2 × 6.2	S3/S2	Excision S3		***November/2007***		
			1.3 × 1.5 × 1.3	S2	Excision S2		September/2010	33	
6	60	M	7.1 × 5.2 × 7.4	S4/S3	Excision S3		***January/2004***		
			5.9 × 7.3 × 5.0	S2	Debulking		July/2007	30	
			4.3 × 3.1 × 2.7	S1	Total sacrectomy	PDS L3-Ilium	December/2008	16	Massive bleeding
7	72	F	17 × 20 × 17	S2/L5	Excision (S2)		***July/2009***		Rectal tear
			5.2 × 6.4 × 5.3	Rt S2	No surgery			4	
			5.7 × 6.5 × 6.7	Lt acetabulum					

Age represents age at the time of diagnosis; mass size is shown in centimeters. *m* months, *Yr* year. Bold and italics indicate first surgery

*Center/proximal vertebral level

^+^Small satellite nodule

**Table 2:** Case details of spinal chordoma and metastasis

Case	Age	Sex	Mass Size (*W* × *H* × *L*)	Primary location*	Operation	Reconstruction	Surgery (m/Yr)	Recurrent (month)	Complications
				Recurrent site					
Spine									
8	35	M	6.0 × 8.8 × 7.6	C2-C3/C1	Laminectomy C1-C3	Screw C1-C4	***May/2016***		
					Corpectomy C2-C3	Cement			
					Extend C2 corpectomy	Cement	August/2016	3	
Metastasis									
9	60	M	3.4 × 4.4 × 3.1	S4	Excision S3		***November/2009***		
			1.7 × 1.7 × 1.7	L2	En-bloc resection	PDS T11-L4, mesh	April/2014	52	
			1.3 × 1.3 × 1.3	T9	En-bloc resection	PDS to T7, mesh	February/2016	21	SCI (recovery)
			3.6 × 4.5 × 3.6	Rt shoulder	Wide excision	Endoprosthesis	November/2018	32	
			5.4 × 6.4 × 5.8	S2/S1 (Lt SI joint extend)	Total sacrectomy	PDS to Ilium; fibular graft	February/2019	3	
10	47	F	8.6 × 9.6 × 7.6	S5	Excision S5		***June/2013***		
			2.1 × 1.3 × 1.8	L4	En-bloc resection	PDS L2-S1,Mesh	July/2013		

Age represents age at the time of diagnosis; mass size is showed in centimeter. *m* months, *Yr* year. Bold and italics indicate first surgery. Case No. 10 revealed L4 metastatic foci at first visit; the second surgery was stage procedure

*Center/proximal vertebral level

### Surgical treatment

#### Sacral chordoma

**Fig. 2:**
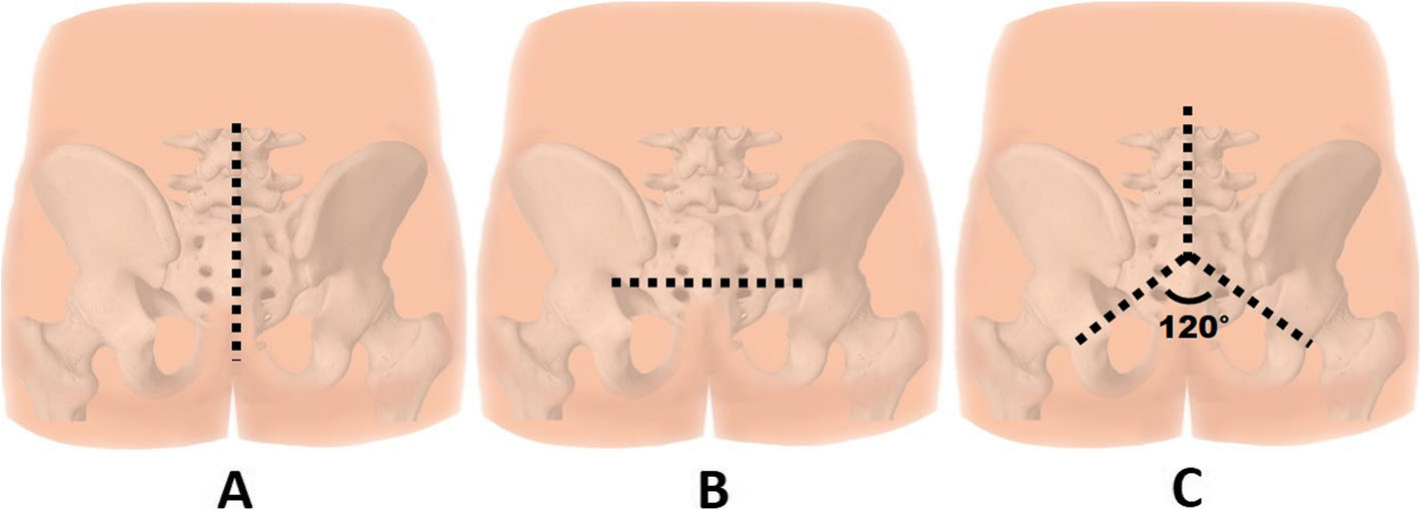
Illustrated skin incision used in the present study. The longitudinal incision (**a**), transverse incision (**b**), and inverted Y incision (**c**). Note: The angle between 2 distal incisions was 120° to avoid skin edge problems

All sacral chordoma was resected by a posterior-only approach [10] after preoperative embolization, except for case No. 2. The skin incisions were either transverse, longitudinal, or inverted Y incision [11] as appropriated (Fig. 2). The superior part of the dissection was at lumbar level or S1 posterior surface regarding of the preoperative planning, if the resection margin was below S2 level the superior dissection margin would end at S1. On the other hand, the dissection would end at L3 if total sacrectomy was planned. The ilio-lumbar instrumentation was performed only in the total sacrectomy cases.

**Fig. 3:**
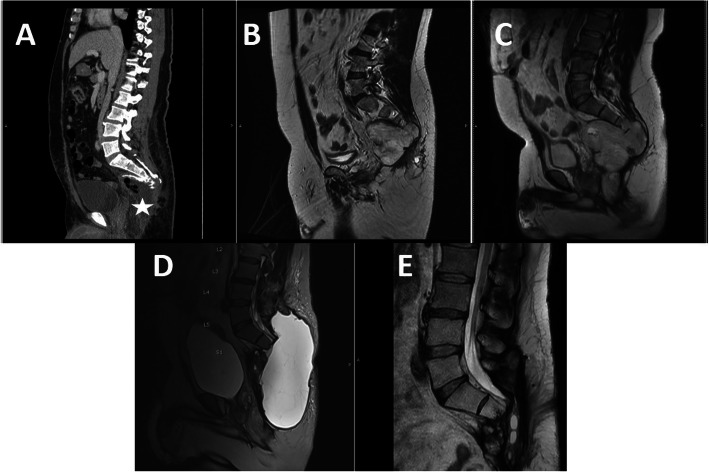
Illustrated surgical result of case No. 2. The initial MRI scan as shown in Fig. 1 in axial (Fig. 1a) and sagittal (Fig. 1b) CT image after surgery (**a**) demonstrated residual tumor at S4 (star). The residual tumor was gradual increase in size as shown in MRI images (**b**, **c**). The surgical removal of remaining mass was performed and revealed large seroma after surgery (**d**) which gradually resolved at 21 months, postoperatively (**e**)

For case No. 2, the patient was a 45-year-old male who presented with gut obstruction from enlarged tumor arising from the pelvis. An exploratory laparotomy, diverting colostomy and subtotal excision (debulking) was performed by abdominal surgeon in 2015. The remaining mass was enlarged and 3 years later, the patient was referred to our department. We performed the second surgery, S3 sacrectomy with tumor removal in 2018 by posterior approach. No serious complication was encountered, only large seroma which gradually resolved. No recurrent tumor was detected throughout the study period (Fig. 3).

In recurrent chordoma, the previous incision was used if it is feasible for the tumor location. The recurrent mass was identified and excised with bony attached, and again, reconstruction procedure was performed if total sacroiliac joint was resected or in the condition with total sacrectomy was necessary. In primary or subsequence resection, if the tumor was ruptured or spilled, copious amounts of normal saline irrigation was performed to decontaminate the tumor as much as possible.

#### Spinal chordoma

**Fig. 4:**
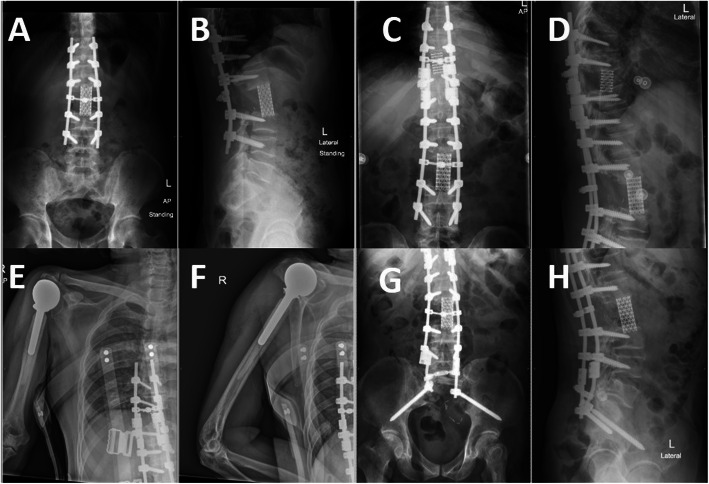
Illustrated oncologic result of case No. 9. The initial surgery was sacrectomy (S3), and 5 years later L2 metastasis was found; therefore, en-bloc spondylectomy was performed (**a**) and (**b**). Two years later T9 metastasis was detected and en-bloc spondylectomy at T9 vertebra was performed with rod extension (**c**, **d**). Three years later, wide excision of tumor with endoprosthesis replacement was performed due to proximal humerus metastasis (**e**, **f**) and total sacrectomy with extended distal fixation to ilium was performed at 3 months, subsequently (**g**, **h**)

Regarding spinal chordoma, at lumbar levels (L2 and L4 vertebrae), the total en-bloc spondylectomy was performed with combined posterior-anterior approach [12]. In the thoracic spine (T9 vertebra), the en-bloc spondylectomy was performed via posterior-only approach, with extended spinal fixation [13] (Fig. 4).

**Fig. 5:**
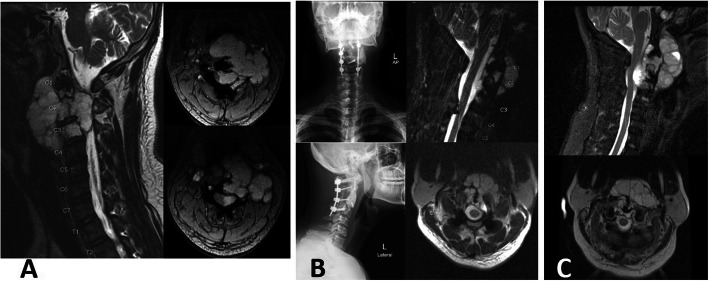
Illustrated oncologic outcome of case No. 8. A 35-year-old male presented with chordoma at C1-C3 with compressive myelopathy, initial MRI (**a**). The surgery was performed by decompressive laminectomy, tumor removal with cervical fixation from C1-C4 and anterior subtotal tumor removal but remaining residual tumor (**b**). The radiotherapy was delayed and resulted in enlargement of the tumor (**c**)

We performed the staged surgery for chordoma at cervical spine, case No. 8. The first stage procedure was performed by the posterior approach. Laminectomy at C1-C3 with C1-C4 fixation was performed, with partial removal of the tumor. After that, we performed the second stage procedure, 1 week later, when the patient status was stable. The anterior approach with partial corpectomy with cement augmentation of C2-C3 was performed by piecemeal fashion with sparing of the den. Unfortunately, because the large amount of tumor still remained, the surgery was performed again at 3 months after the second operation (Fig. 5). However, the oncologic outcome is not favorable.

### Follow-up

Postoperative radiotherapy was performed in every case that revealed tumor contamination caused by rupture of mass, positive tumor margin either in an intraoperative direct observation, or pathologic examination report. The oncologist was consulted for further evaluation in every case. Per our routine, the patients were followed at 2 weeks postoperatively for surgical wound examination, and re-explored if the surgical-site infection was suspected. Then, follow-up at 1 to 3 months and then every 6 months with a radiograph of the spine, pelvis or sacrum as appropriated. The MRI or CT scan was performed every 6 months until 1 year postoperatively, and then every year. A physical examination was performed at every single visit and if the recurrent tumor was suspected, such as new onset of pain or palpable mass, the investigation with MRI or CT scan was immediately performed.

### Oncologic outcomes

The recurrence site was almost at the sacrum (osteotomy site) and adjacent bone, due to positive surgical margin or tumor contamination at the time of first surgery, the recurrence in surrounding muscle and surgical bed was found in 3 patients. The average time from first index surgery to first episode of recurrent tumor detection or metastasis, except that of case No. 8 which was incomplete treatment, was 27 months (range, 4–52 months) postoperatively and the average time from second index surgery to second episode of recurrent tumor detection was 16 months (range, 7–21 months). The average number of recurrences was 1.4 (range, 1–2), excluding those with metastasis. Result from 5-year survival analysis, including metastasis, using Kaplan-Meier survival analysis, showed the median time for the first recurrent was 30 months with 95% confident interval range from 4 to 52 months (except that of case no 8). Two patients were deceased in an attempt of resection for recurrent tumor due to massive blood loss. As of last follow-up, three patients live with the disease and three patients encountered recurrence, but the tumors were inoperable. Only 2 patients were disease-free at latest follow-up. There were 2 patients with confirmed distant metastases. The mean follow-up time was 5.6 years (range 1–13 years) (Table 3).

**Table 3:** Oncologic outcome

Case	Age	Sex	Primary location*	Oncologic outcome	First visit	Follow-up
Sacrum						
1	62	M	S4/S3	Live with disease	2014	Present
2	45	M	S4/L4	Disease free	2015	Present
3	57	F	S4/S2	Live with disease^+^	2009	Loss F/U 2017
4	54	M	S3/S2	Perioperative dead	2010	
5	45	F	S3/S2	Disease free	2007	Present
6	60	M	S4/S3	Perioperative dead	2004	
7	72	F	F	Extensive mass recurrent; inoperable	2009	Loss F/U 2010
Spine						
8	35	M	C2-C3/C1	Extensive mass recurrent; inoperable	2016	Loss F/U 2017
Metastasis						
9	60	M	S4	Live with disease	2009	Present
10	47	F	S5 and L4	Multiple metastasis; inoperable	2013	Loss F/U 2014

Age represents age at the time of diagnosis

*Center/proximal vertebral level

^+^Data at last follow-up

## Discussion

Chordoma is a rare tumor with difficult to manage. It can appear at any location along axial skeleton. The sacrococcygeal region is the most common site, accounting for 65% of all cases of chordomas, followed by the spheno-occipital/nasal (25%), cervical (10%), and thoracolumbar (5%) spines [14]. Because of the slow growing rate and the often nonspecific nature of symptoms, chordoma often appears to be an enlarged mass at the time of presentation [15]. Boriani et al. has reported that the slow and gradual onset of pain is the most consistent complaint [16]. The time from onset to diagnosis has been reported range from 4 to 24 months [17]. Chordoma is considered as poorly-responsive tumor to conventional radiotherapy and chemotherapy. Thus, surgical resection remains the mainstay of treatment. The oncologic outcomes in term of local control and overall survival are associated with the ability to perform radical resection [18, 19]. However, because of the extensive lesion and nearby vascular or neural structures cause margin-free en-bloc surgery is difficult to perform. Moreover, because the tumor capsule is thin therefore violation of the capsule is sometimes unavoidable and result in contamination of tumor in the operative field and end-up with local recurrent [20].

Although the advancements of surgical techniques have been developed, the consensus on the optimal surgical resection remains unclear. To perform en-bloc margin-free excision, many authors recommend a combined approach [20, 21]. However, posterior-only approach for en-bloc resection of sacral chordoma has been established with favorable outcome [10, 22]. In our series, we performed the surgery of sacral chordoma by posterior-only approach but in different incision, the longitudinal incision is used in almost cases because of lower risk of wound complication and easy to extend incision proximally if necessary. The transverse incision has a benefit to reach the ilium and sciatic notch without extensive dissection and prefer to use in low sacral resection. The inverted Y incision has the highest risk of wound complication, but this incision has combined the advantage of both longitudinal and transverse incision; this incision is used when the total sacrectomy is planned.

Although local recurrence is common following surgical treatment of spinal chordoma, reported in the literatures ranging from 19 to 54% [23]. This may be explained by the difference in severity and invasion to nearby structures that preclude the en-bloc resection to be possible. In this series, all patients were referred to our institution, which may be delayed in diagnosis so further enlarged tumor mass with more extensive invasion makes it difficult to prevent tumor capsule violation or sometimes impossible for en-bloc margin-free resection. The large size of tumor at presentation and the complexity of sacral structures might partially explain the high rate of local recurrence [24]. Furthermore, we prefer to save the sacral roots and bony structure as much as possible to conserve quality of life of patients after the surgery, resulting in positive surgical margin in all cases; this may result in recurrent disease due to relatively low response to radiotherapy [25], although time to local recurrent may not directly relate to the surgical margin as demonstrated in a large retrospective study [24]. Result from this study also showed that chordoma is most likely recurrent on the remaining stump, surgical base, and adjacent soft tissue such as gluteal muscle comparable with previous studies [4, 16, 18]. According to this finding, en-bloc margin-free surgical resection is mandatory to prevent recurrence and surgeons should meticulously seek for local tumor seeding especially when the tumor is rupture. In addition, the anterior approach to free the vascular and vital structures and manage engorged pelvic venous plexus before posterior resection of the sacral tumor should be performed especially in the recurrent large sacral chordoma to prevent excessive bleeding or damage of important structures form scar tissues.

**Fig. 6:**
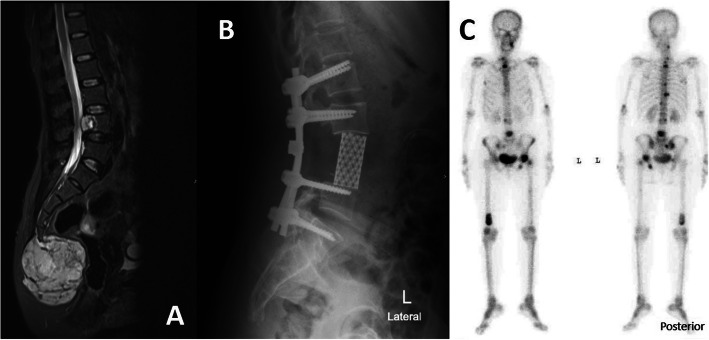
Illustrated oncologic result of case No. 10. A 47-year-old female presented with concomitant chordoma at distal sacrum and L4 vertebra (**a**), the sacral mass and L4 vertebra were removed by en-bloc resection within 1 month after presentation (**b**). However, subsequent bone scan revealed generalized bone metastasis (**c**)

The present study also assessed the surgical outcome of mobile spinal chordoma. The local recurrence after resection of spinal chordoma was not encountered after en-bloc spondylectomy, case No. 9 and 10, but we were not able to prevent distant metastasis [24] (Fig. 6). However, in cervical spine chordoma (case No. 8), this study found that recurrence in surrounding soft tissue was encountered. Although the en-bloc surgical resection of the upper cervical spine is not feasible and gross tumor piecemeal resection could provide acceptable long-term survival up to 3 years [26, 27]. In this patient, the adjuvant radiotherapy was delayed and may lead to rapid local recurrence.

With the high rate of recurrence, intensive follow-up is necessary after initial resection. Daniel et al. proposed the protocol of follow-up chordoma after resection as follows: patients undergo CT scan of the resection bed immediate postoperatively, and MRI scanning is performed within 48 h. After release from the hospital, surveillance MRI scans are obtained every 3 months in the first year following resection, every 6 months in the second year, and annually thereafter [28]. Periodic chest X-Ray and whole body scan have also been purpose in many studies to detect the distance of metastasis [2, 4, 24].

Radiotherapy can be used as an adjuvant treatment for chordoma with incomplete resection or positive margins; however, the relative radioresistance and proximity to sensitive neurologic tissues and other intrapelvic organs make chordoma difficult to treat with standard radiation therapy [8, 29]. Conventional photon-beam radiotherapy is commonly used as an adjuvant treatment in patients undergoing subtotal excision. However, reports vary as to whether additional survival benefit is derived. Conventional treatments with doses of 40 to 60 Gy reported 5-year local control rates of 10 to 40% [29].

To date, proton beam therapy makes use of protons or charged particles such as carbon ions, helium, and neon. This technology can deliver high-dose radiation to the target tissue that, in principle, could surpass even the most sophisticated photon radiation delivery techniques while minimizing damage to nearby sensitive structures and demonstrated as a promising treatment modality for chordoma [29, 30].

There were some limitations in this study. First, this study was slightly small in number of patients due to the extremely rare disease. Second, because of long-term follow-up period, some patients had loss to follow and there were some missing follow-up data. Third, the MRI or CT scan was not performed immediately after radiotherapy; therefore, we could not confirm the remaining residual tumor. Larger-scale prospective study from multiple centers should be conducted to provide more accurate results.

In conclusion, en-bloc free-margin resection is mandatory to prevent recurrence of chordoma. Early adjuvant radiotherapy seems to provide benefit if margin-free resection is not achieved. The clinical vigilance and investigation to identify tumor recurrence should be performed every 3 to 6 months, especially, in the first 30 months and annually thereafter. Detection of recurrence in the early stage with a small mass may be the best chance to perform an en-bloc margin-free resection to prevent further recurrence.

## Data Availability

The datasets used and analyzed during the current study are available from the corresponding author on reasonable request.
